# EMG features dataset for arm activity recognition

**DOI:** 10.1016/j.dib.2025.111519

**Published:** 2025-04-04

**Authors:** Koundinya Challa, Issa W. AlHmoud, Chandra Jaiswal, Anish C. Turlapaty, Balakrishna Gokaraju

**Affiliations:** North Carolina A&T State University, 1601 E Market St, Greensboro, NC 27411, United States

**Keywords:** Electromyography sensors, Hand gestures, Machine learning, Classification

## Abstract

This study presents a dataset on hand gesture recognition using electromyography (EMG) signals. The data was collected from eight healthy subjects aged between 19 and 35 years, with each subject performing three distinct hand gestures (lifting, grabbing, and flexing). Surface EMG signals were recorded using the Delsys Trigno Wireless biofeedback system from four sensors placed on the dominant hand's Palm A, Palm B, Biceps, and Forearm. The signals were sampled at 2000 Hz and segmented into gesture trials for analysis. The raw EMG data were filtered and processed to extract seven time-domain features across each channel, resulting in 28 total features. These features were reduced using Principal Component Analysis (PCA) to six components, which accounted for 95 % of the variance. The dataset was then used to train and test machine learning models (Random Forest and Logistic Regression) for gesture classification. This dataset has potential reuse in developing gesture recognition algorithms, enhancing prosthetic control, or exploring human–computer interaction (HCI) applications.

Specifications Table

**Table:** 

Subject	Wireless EMG Sensor Data.
Specific subject area	Sensor Technology.
Type of data	Raw, Analyzed, Filtered, Processed
Data collection	Surface electromyography (sEMG) signals were collected from eight healthy subjects using the Delsys Trigno Wireless biofeedback system. Four EMG sensors were placed on the dominant hand (Palm A, Palm B, Biceps, and Forearm). Signals were sampled at 2000 Hz with <750 nV noise. Subjects performed lifting, grabbing, and flexing gestures, each repeated for 25 s, followed by 10 s of rest. Data were digitally filtered (20–500 Hz) to remove noise. Principal Component Analysis (PCA) was used for feature selection.
Data source location	Raw EMG sensor data was collected and stored in an Excel file, and the eventually extracted to a csv file.
Data accessibility	Repository name: EMG DATASET Data identification number: 10.5281/zenodo.13882683 Direct URL to data: https://tinyurl.com/yd8ujupj
Related research article	K. Challa, I. W. AlHmoud, A. K. M. Kamrul Islam and B. Gokaraju, ``EMG-Based Hand Gesture Recognition Using Individual Sensors on Different Muscle Groups,'' 2023 IEEE Applied Imagery Pattern Recognition Workshop (AIPR) , St. Louis, MO, USA, 2023, pp. 1–4, doi: 10.1109/AIPR60534.2023.10440702 [1].

## 1 Value of the Data

• Comprehensive EMG Signal Data: The dataset offers a comprehensive collection of EMG signals captured from four different muscle groups, providing valuable insights into muscle activations during various hand gestures. This level of detail enables researchers to analyze complex muscular patterns, making it an important resource for studies in biomechanics, motor control, and rehabilitation
• Diversity in Hand Gestures and Subjects: The dataset includes recordings from eight subjects performing three distinct hand gestures (lifting, grabbing, and flexing). This diversity makes the data applicable to a range of studies focused on understanding inter-individual variability in gesture recognition, aiding in developing more generalized EMG-based models.
• Machine Learning Application Potential: The dataset's structure, featuring both raw and processed EMG signals with extracted features, makes it ideal for training and evaluating machine learning algorithms. It serves as a valuable resource for researchers aiming to develop or benchmark gesture recognition models, providing a foundation for improving classification accuracy.
• Human–Computer Interaction (HCI) Research: Given the dataset's focus on capturing natural hand movements, it holds potential for advancing HCI applications, such as developing intuitive gesture-based interfaces for virtual reality, robotics, or prosthetic devices.
• High-Frequency Signal Sampling: The EMG signals were sampled at a high frequency (2000 Hz), ensuring a detailed capture of muscle activities. This high-resolution data allows for more precise analysis of gesture dynamics, making it valuable for researchers who require detailed signal characteristics for feature extraction and analysis.

## 2 Background

The original motivation for compiling this dataset was to explore and develop a reliable method for hand gesture recognition using electromyography (EMG) signals. Hand gestures play a significant role in human–computer interaction (HCI) and prosthetic control, where accurately interpreting muscle activity can lead to more intuitive and responsive systems [2,3]. Theoretical considerations were based on the idea that surface EMG signals, captured from different muscle groups, could provide a rich source of information about various hand movements [4,5]. Previous studies have shown that features extracted from EMG data can serve as effective inputs for machine learning algorithms to recognize gestures and control robotic or prosthetic systems [6,7]. Methodologically, individual sensors were placed on different parts of the hand and arm to obtain multi-channel EMG data, enabling the capture of subtle muscle activations associated with distinct gestures [8,9].

The dataset was generated as part of a broader research effort to compare the performance of machine learning algorithms, such as Random Forest and Logistic Regression, in classifying these gestures. This data article extends the original research by providing detailed information about the dataset, feature extraction methods, and data collection process [[10], [11], [12]]. This makes it accessible for reuse in future studies related to gesture recognition, EMG analysis, or machine learning applications in HCI and rehabilitation [12].

## 3 Data Description

The dataset contains 2, 205 observations with 5 features, representing electromyography (EMG) signals from different muscle locations. Below is a brief description of each feature:

1. **EMG_1_A_Palm_front**: EMG signals recorded from the front of the palm, with values ranging from -0.0009026 to 0.001954. The mean value is close to zero, indicating minimal muscle activity on average.
2. **EMG_1_B_Palm_back**: Captures signals from the back of the palm, with values ranging from -0.006863 to 0.002434. The standard deviation shows moderate variability in the signals.
3. **EMG_2_Forearm**: EMG signals from the forearm, with minimal deviation from the mean. The values range between -0.000031 and zero.
4. **EMG_3_Bicep**: Signals from the bicep muscle with a similar range as the forearm, showing subtle variations in muscle activity.
5. **Class**: This categorical feature labels each observation into three classes, evenly distributed with 735 instances for each class, representing different states of muscle activity.

## 4 Experimental Design, Materials and Methods

Participants: Eight healthy subjects (5 males and 3 females), aged 19–35, participated in the study. Prior to the experiment, each subject provided written consent. Subjects were categorized by sex and dominant hand (6 right-handed, 2 left-handed).

Instrumentation: Surface EMG signals were acquired using the Delsys Trigno Wireless biofeedback system, a high-fidelity EMG acquisition device. Four EMG sensors were used, with a sampling frequency of 2000 Hz and a baseline noise level of <750 nV. Each sensor was capable of capturing EMG signals from different muscle groups accurately.

**Fig. 1:**
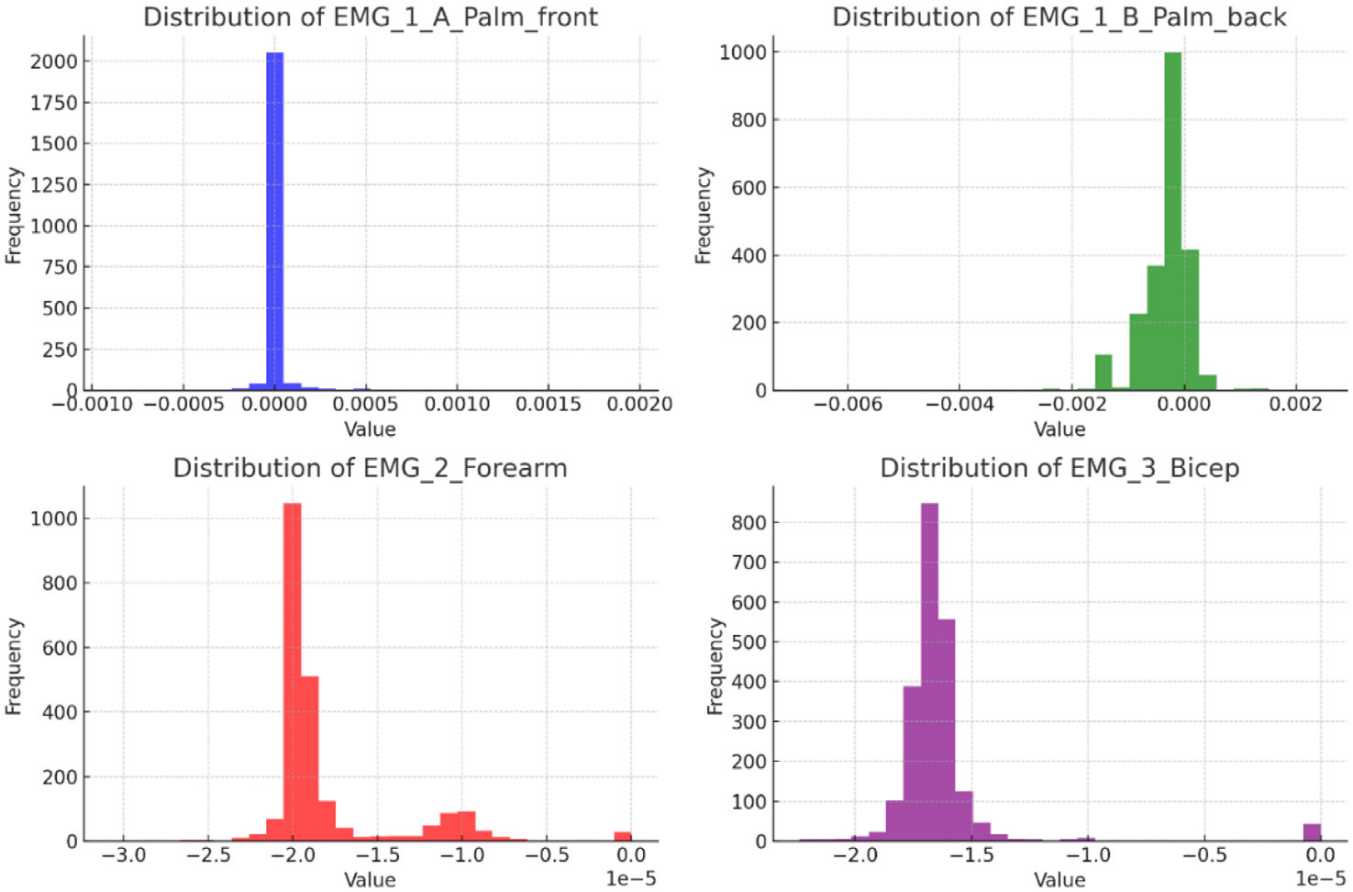
Histograms of the features.

**Fig. 2:**
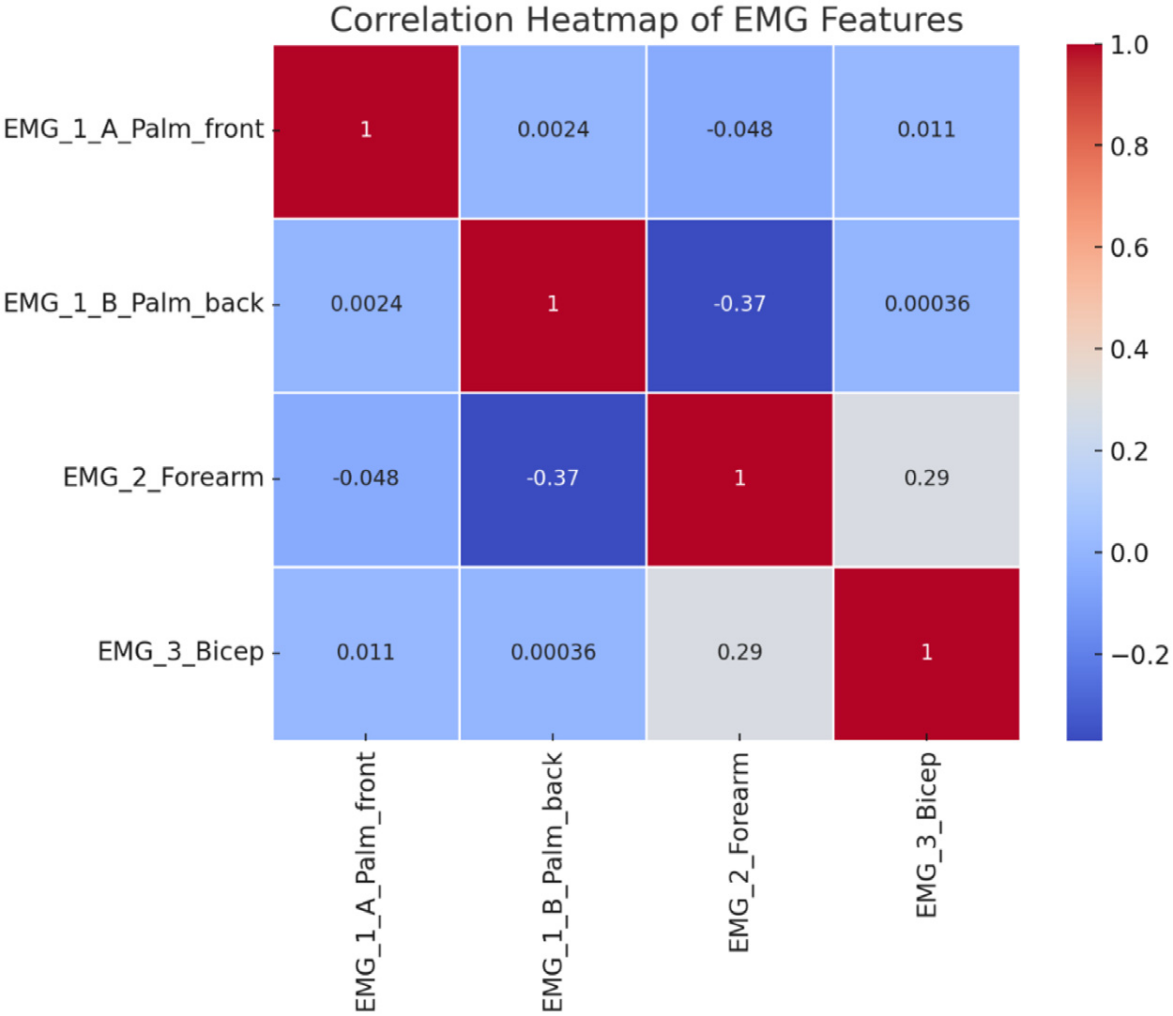
Heat map of the features.

Sensor Placement: The four EMG sensors were placed at the following locations on the dominant hand:

The histograms and correlation matrix of the investigated features are shown in Fig. 1, Fig. 2.

• Palm A
• Palm B (opposite sides of the palm)
• Biceps
• Forearm

**Fig. 3:**
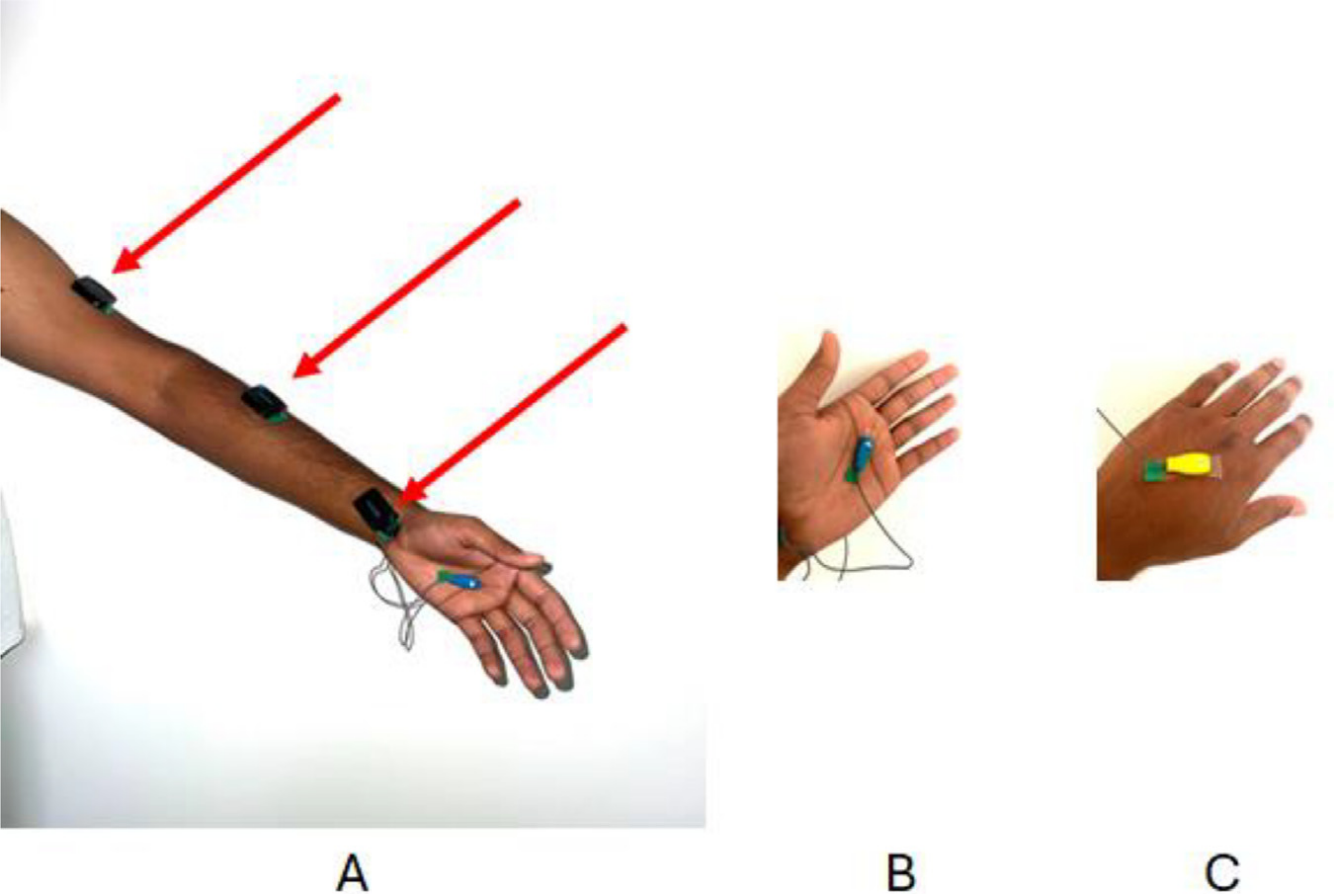
Sensors placement. A) forearm and biceps, B) Palm A, C) Palm B. [1].

Fig. 3 depict the sensor placements.

The placement ensured comprehensive coverage of the muscle groups involved in hand gestures.

**Fig. 4:**
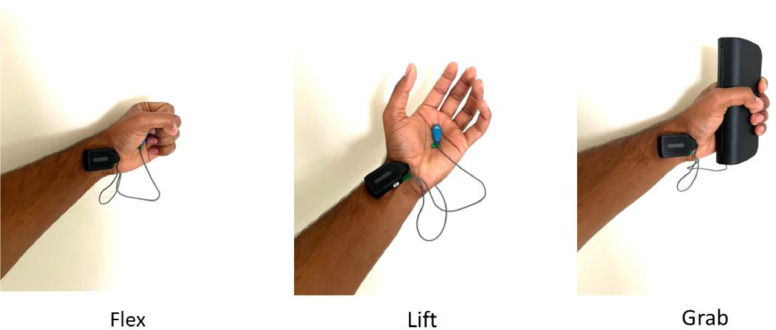
Investigated hand gestures [1].

Hand Gestures: Each subject performed three distinct hand gestures (Fig. 4):

• Lifting
• Grabbing
• Flexing

Subjects repeated each gesture continuously for 25 s, followed by a 10-s rest period. This process was repeated three times per gesture to collect sufficient data.

### 4.1 Data collection protocol

The experiment was conducted in a university student laboratory.

Before attaching the sensors, the subject's dominant hand was cleaned using sanitizing wipes to improve signal quality.

The total recording time per subject was approximately 10 min.

### 4.2 Data preprocessing

The acquired raw EMG signals underwent a bandpass filter (20–500 Hz) to remove noise and motion artifacts.

Signals were then segmented into individual gesture trials for further analysis.

Feature Extraction: Seven time-domain features were extracted from each of the four EMG channels using Python code. The features were:

• Integrated EMG (IEMG)
• Simple Square Integral (SSI)
• Mean Absolute Value (MAV)
• Root Mean Square (RMS)
• Waveform Length (WL)
• Willison Amplitude (WAMP)
• Willison Amplitude with Variance (WAMPV)

This resulted in 28 total features per gesture trial.

### 4.3 Noise and motion artifact removal

The raw EMG signals were preprocessed to enhance signal quality by applying a bandpass filter (20–500 Hz). This effectively eliminates motion artifacts and high-frequency noise while preserving essential gesture-related signal components. Additionally, baseline noise levels were measured at <750 nV, ensuring high-fidelity data acquisition.

### 4.4 Dimensionality reduction

Principal Component Analysis (PCA) was employed to reduce the dimensionality of the extracted features while retaining 95% of the variance. The selection of six principal components was determined based on the cumulative explained variance method. A comparative analysis of using different numbers of components showed that reducing beyond six led to significant loss of discriminative power, negatively impacting classification accuracy. Conversely, adding more components beyond six resulted in minimal improvement while increasing computational complexity.

### 4.5 Software and tools used

Data collection: Delsys EMGWorks software.

Data analysis and processing: Python with libraries such as numpy, pandas, and scikit-learn.

Feature extraction and classification modeling: Python-based scripts using scikit-learn.

### 4.6 Outcomes and future scenarios

This dataset has significant implications in multiple domains, including prosthetic control, rehabilitation, and human–computer interaction (HCI). Future research can leverage this dataset to develop robust machine learning models for real-time gesture classification, enabling applications such as assistive devices for individuals with motor impairments. Additionally, the dataset can be integrated into virtual reality (VR) and robotics to facilitate more intuitive control mechanisms based on EMG signal interpretation. Further extensions of this work could involve collecting data from individuals with neuromuscular disorders to enhance the dataset's applicability in medical rehabilitation.

## Limitations

**Limited Variety of Hand Gestures**: The dataset may be restricted in terms of the variety of hand gestures it captures. If only a few specific gestures are recorded, it might not provide sufficient data to train models for broader applications, such as recognizing complex or subtle hand movements. This could limit its usability in real-world scenarios requiring diverse gesture recognition.

## Ethics Statement

This study involved human subjects, and informed consent was obtained from all participants prior to data collection. The research was conducted in accordance with the Declaration of Helsinki, and the experimental protocol was approved by the relevant Institutional Review Board (IRB) with approval number (21-0122) at North Carolina Agricultural and Technical State University. All procedures, including participant recruitment, data collection, and handling, adhered to ethical guidelines, ensuring participant safety, privacy, and confidentiality throughout the study. The subjects were informed about the purpose, risks, and benefits of the study, and participation was voluntary.

## Credit Author Statement

**Koundinya Challa:** Conceptualization, Methodology, Data curation, official draft preparation, **Chandra Jaiswal:** Reviewing and Editing, **Issa W. AlHmoud:** Supervision, Review & Editing, **Anish C. Turlapaty:** Supervision, Validation, **Balakrishna Gokaraju:** Funding acquisition, resources.

## Data Availability

Google DriveEmg Sensor Dataset (Original data).

## Appendix Supplementary materials

Image, application 1
