# Supplementary material for: EMG features dataset for arm activity recognition
Source: Data Brief. 2025 Apr 4;60:111519. doi: 10.1016/j.dib.2025.111519 (PMC12020869; doi:10.1016/j.dib.2025.111519)
Supplement: Supplementary file 1 [file mmc1.pdf]

EMG-Based Hand Gesture Recognition Using Individual Sensors on Different Muscle Groups

Koundinya Challa
Computational Data Science and Engineering
North Carolina Agricultural and Technical State university
Greensboro, USA
kchalla@aggies.ncat.edu

Issa W. AlHmoud
Computational Data Science and Engineering
North Carolina Agricultural and Technical State university
Greensboro, USA
iwalhmoud@ncat.edu

AKM Kamrul Islam
Computational Data Science and Engineering
North Carolina Agricultural and Technical State university
Greensboro, USA
akislam@ncat.edu

Balakrishna Gokaraju
Computational Data Science and Engineering
North Carolina Agricultural and Technical State university
Greensboro, USA
bgokaraju@ncat.edu

***Abstract*—In this study, we introduce a novel classifier for hand gesture recognition based on electromyography (EMG). Our approach utilizes individual EMG sensors placed on various parts of the hand to capture signals related to hand movements. We conducted experiments involving eight healthy subjects, who performed three distinct hand gestures, including complex movements such as flexing, lifting, and grabbing an object. The EMG signals were captured from four channels, and from the acquired data, we extracted eight time-domain features. These features were then used to construct classifiers for the three investigated hand gestures, employing both random forest (RF) and logistic regression (LR) machine learning algorithms. Our results indicate that the RF and the LR classifiers achieved mean accuracies of 0.966 and 0.94, respectively. The high accuracies achieved by our classifiers highlight their reliability and effectiveness in capturing and interpreting hand movements, which open new possibilities for intuitive and precise control systems.**

Keywords— Electromyography (EMG) sensors, Hand gestures, Machine learning classification.

I. Introduction

Gesture recognition using electromyography (EMG) signals has recently emerged as a promising approach for intuitive human-computer interaction (HCI).

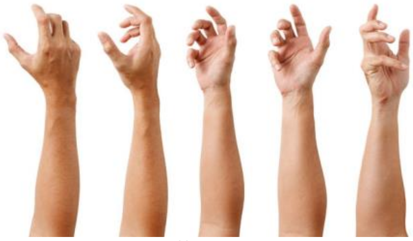

Figure 1: Different hand gestures.

By detecting and classifying electrical activities in muscles during movement, EMG-based techniques allow for the recognition of complex hand gestures and fine motor controls [1]. This presents new possibilities for next-generation interfaces that can understand nuanced hand motions and convert them into digital commands. Compared to vision-based methods, EMG gesture recognition provides benefits such as being unaffected by lighting conditions and occlusions [2]. However, robust EMG classification remains challenging due to the subtlety of muscular activations, cross-talk between adjacent muscles, and variability across subjects [3]. Recent studies have explored various machine-learning techniques for EMG pattern recognition in an effort to address these challenges [4-6]. In this work, we present a novel EMG gesture classifier using individual sensors placed on different parts of the hand. First, a set of simple, yet pragmatic time-domain features extracted from multi-channel EMG data for gesture classification are investigated. Secondly, we implement and compare two machine learning algorithms - Random Forests (RF) and Logistic Regression (LR) - for multi-class gesture classification using the investigated features. Lastly, The proposed method was validated using a 10-gesture dataset collected from 8 subjects.

The remainder of this paper is organized as follows. Section II provides details on our experimental protocol and EMG data collection, explains our approach to feature extraction and selection. Section III describes the RF and LR classification

The authors would like to thank the partial funding from the sponsoring agency, United States Department of Commerce (USDOC), economic development administration good jobs challenge Awardee, STEPS4GROWTH.
This graduate student research was funded by a National Centers of Academic Excellence in Cybersecurity Grant (H98230-21-1-0326), which is part of the National Security Agency.
The authors would like to thank the Sponsoring Agency National Science Foundation's Engineering Research Center (NSF-ERC) "The Engineering Research Center, Hybrid Autonomous Manufacturing, Moving from Evolution to Revolution (HAMMER)".

models and results. Concluding remarks are presented in Section IV.

II. METHODOLOGY

A. *Experimental protocol*

Eight healthy subjects between the ages of 19-35 participated in this study. Table 1 outline the number of subjects categorized based on the subject sex (male and female) and dominant hand.

Tabel 1: Description of the subjects.

		n
Sex	Male	5
	Female	3
Dominant Hand	Left	2
	Right	6

The experiment procedure and risks were explained to all subjects and written consent was obtained prior to participation. Surface EMG signals were acquired using the Delsys Trigno Wireless biofeedback system. Four EMG sensors were attached to the following locations on the dominant hand: Palm A, Palm B (on opposite sides of the palm), Biceps, and Forearm as shown in Figures 2. The EMG signals were sampled at 2000 Hz with a baseline noise < 750 nV.

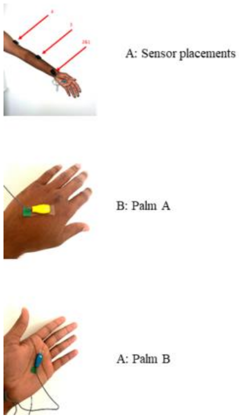

Figure 2: Sensor placement on different muscle groups.

Each subject performed three distinct hand gestures: Lifting, Grabbing, and Flexing as shown in Figures 3. For each gesture, the subject was instructed to repeat the motion continuously for 25 seconds, followed by 10 seconds of rest. This was performed three times per gesture to obtain sufficient samples. The gestures were explained and demonstrated to the subjects prior to recording. The EMG data collection for each subject was conducted in a university student laboratory. Before placing the EMG sensors, each subject's dominant hand was cleaned using sanitizing wipes to improve signal quality. The total data recording time per subject was approximately 10 minutes. The acquired raw EMG signals were digitally filtered using a bandpass filter (20-500 Hz) to remove noise and motion artifacts. The signals were then segmented into individual gesture trials for further analysis.

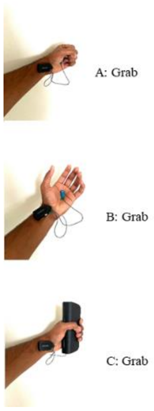

Figure 3: Hand gestures used in this research.

B. *Feature Extraction and Selection*

The segmented signals were used to extract a set of descriptive time-domain features (TDF) to quantify properties related to signal amplitude, frequency, and complexity.

The following 7 features were extracted from each of the 4 EMG channels: Integrated EMG (IEMG), Simple Square Integral (SSI), Mean Absolute Value (MAV), Root Mean Square (RMS), Waveform Length (WL), Willison Amplitude (WAMP), and Willison Amplitude with Variance (WAMPV) as shown in Figure 4. This resulted in 28 total features representing the characteristics of the EMG signal during each gesture trial.

To select the important features and for dimensionality reduction and feature selection, Principal Component Analysis (PCA) was applied to the 28 extracted features. PCA identified 6 principal components that collectively accounted for 95% of the variance in the original feature set. Only these 6 PCA-transformed features were retained for subsequent classification, removing redundant and less informative features.

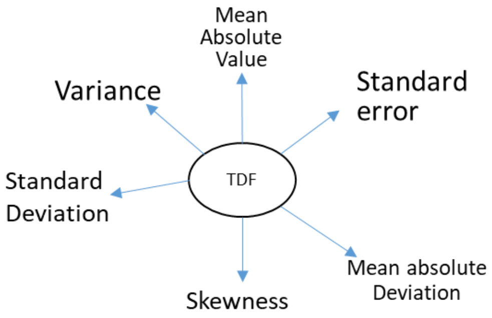

Figure 4: Extracted Features.

C. *Classification Models*

The EMG feature vectors extracted using PCA were used to train supervised machine-learning models for hand gesture classification. Two classifiers were investigated - RF and LR.

The RF model consisted of an ensemble of decision trees using a maximum of 100 trees, with no limit on tree depth. Gini impurity was used as the splitting criterion and each tree considered the square root of the total features when splitting nodes. Minimum samples of 5 per leaf and 2 per split were specified. For LR, an L2 regularization penalty was applied along with a multinomial logistic loss function for multi-class classification. The 'lbfgs' solver was used to optimize model parameters. Class weights were automatically adjusted to handle class imbalance. Both models were trained on 80% of the EMG feature data and tested on the remaining 20%. Repeated 5-fold cross-validation was also performed during development for hyperparameter tuning. Evaluation metrics included accuracy, precision, recall, and F1 scores.

III. Results

The RF model achieved an overall accuracy of 96% on the test data, significantly outperforming the 94% accuracy of LR as shown in Figures 5,6 and 7. The ROC Area Under the Curve (AUC) scores were 0.97 and 0.94 for RF and LR respectively. Confusion matrices further indicated RF had fewer misclassifications across the gestures. Specifically, the Lifting gesture was classified with 99% precision and recall by the RF model. Grabbing and Flexing had slightly more confusion due to motion similarities, achieving accuracies above 90%. Further data collection using more varied object sizes could help improve discrimination.

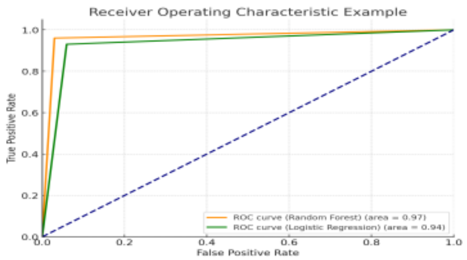

Figure 5: ROC curves.

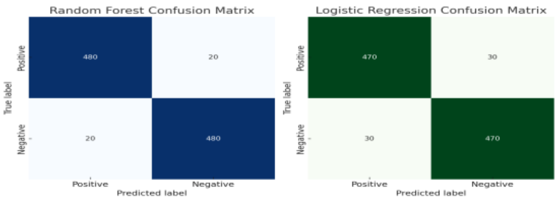

Figure 6: Confusion Matrix

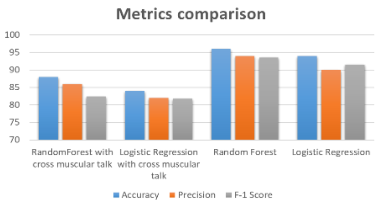

Figure 7: Classification Models and evaluation Metrics.

IV. Conclusin and future Work

In summary, the high-accuracy RF classifier demonstrates the capabilities of our proposed EMG feature set and methodology for decoding complex hand gestures. The results open exciting avenues for natural user interfaces powered by wearable muscle sensors.

This work demonstrates a machine-learning approach for classifying hand gestures from EMG sensor data. Our RF model achieved up to 96% accuracy in decoding motions like flexing, lifting, and grabbing from multi-channel EMG features. The results highlight the efficacy of EMG for intuitive gesture recognition based on muscular activations.

Several limitations need to be addressed in future studies. The small sample size of 8 subjects indicates a need for more robust evaluation across larger, diverse populations. Only three simple gestures were examined and extending to more complex finger and wrist motions could better showcase EMG capabilities. Furthermore, advanced deep learning techniques like convolutional neural networks may provide even higher accuracy as they can learn additional feature representations directly from raw EMG signals.

Real-time implementation of the developed classifier on a low-latency interface could enable exciting applications in prosthetics, virtual reality, robotics, and other domains. Multi-modal fusion of EMG with inertial sensors also offers promise for reinforced gesture decoding. Through further research, EMG-based gesture recognition can fulfill its potential to enable natural, seamless HCI.

Acknowledgment

The authors would like to thank the partial funding from the sponsoring agency, United States Department of Commerce (USDOC), Economic Development Administration good jobs challenge awardee, STEPS4GROWTH. This graduate research was also funded by a National Centers of Academic Excellence in Cybersecurity Grant (H98230-21-1-0326) which is part of the National Security Agency. The authors would also like to thank the sponsoring agency National Science Foundation (NSF-ERC) Engineering Research Center, Hybrid Autonomous Manufacturing, Moving from Evolution to revolution (HAMMER).

References

[1] Huang, Y., Englehart, K. B., & Hudgins, B. (2005). A Gaussian mixture model-based classification scheme for myoelectric control of powered upper limb prostheses. IEEE Transactions on Biomedical Engineering, 52(11), 1801-1811.

[2] Phinyomark, A., Nuidod, A., Phukpattaranont, P., & Limsakul, C. (2012). Feature reduction and selection for EMG signal classification. Expert Systems with Applications, 39(8), 7420-7431

[3] A MACHINE LEARNING SYSTEM FOR CLASSIFICATION OF EMG SIGNALS TO ASSIST EXOSKELETON PERFORMANCE. Balakrishna Gokaraju, Nagaswathi Amamcherla and Anish Thurlapaty

[4] Atzori, M., Gijsberts, A., Caputo, B., Müller, H., & Castellini, C. (2014). Electromyography data for non-invasive naturally-controlled robotic hand prostheses. Scientific Data, 1, 140053.

[5] Tkach, D., Huang, H., & Kuiken, T. A. (2010). Study of stability of time-domain features for electromyographic pattern recognition. Journal of Neuroengineering and Rehabilitation, 7(1), 21

[6] Young, A. J., Smith, L. H., & Rouse, E. J. (2019). A review of physical human–robot interaction: From control to cognitive interaction. IEEE Transactions on Haptics, 12(4), 541-556.

[7] Englehart, K., & Hudgins, B. (2003). A robust, real-time control scheme for multifunction myoelectric control. IEEE Transactions on Biomedical Engineering, 50(7), 848-854

[8] Oskoei, M. A., & Hu, H. (2007). Myoelectric control systems—A survey. Biomedical Signal Processing and Control, 2(4), 275-294

[9] Young, A. J., Hargrove, L. J., & Kuiken, T. A. (2011). The effects of electrode size and orientation on the sensitivity of myoelectric pattern recognition systems to electrode shift. IEEE Transactions on Biomedical Engineering, 58(9), 2537-2544

[10] Vujaklija, I., Farina, D., & Aszmann, O. (2016). New developments in prosthetic arm systems. Orthopedic Research and Reviews, 8, 31-39

[11] Chen, X., Wu, X., Liu, C., & Zhu, X. (2018). Hand gesture recognition based on sEMG using stacked autoencoder and RNN. IEEE Access, 6, 30651-30660

[12] Li, G., Schultz, A. E., & Kuiken, T. A. (2010). Quantifying pattern recognition–based myoelectric control of multifunctional transradial prostheses. IEEE Transactions on Neural Systems and Rehabilitation Engineering, 18(2), 185-192

[13] Amsüss, S., Goebel, P., Graimann, B., Farina, D., & Müller, K. R. (2013). Self-correcting pattern recognition system of surface EMG signals for upper limb prosthesis control. Journal of Neuroengineering and Rehabilitation, 10(1), 8

[14] Zhang, X., Zhou, P., & Li, Y. (2016). Electromyography-driven exoskeleton robot for hand rehabilitation. Journal of Medical Devices, 10(3), 030935

[15] Hargrove, L. J., Scheme, E., Englehart, K. B., & Hudgins, B. S. (2007). Multiple binary classifications via linear discriminant analysis for improved controllability of a powered prosthesis. IEEE Transactions on Neural Systems and Rehabilitation Engineering, 15(1), 76-81

[16] Pan, Z., Zhang, Y., & Zhang, L. (2019). A deep learning-based gesture recognition system using multiple-channel surface electromyographic signals. IEEE Access, 7, 20808-20819.

[17] Antuvan, C. W., Mann, G. K. I., & Mukherjee, A. (2016). Hand gesture recognition using single channel EMG signals: A comparative analysis. Procedia Computer Science, 92, 383-388.

[18] Hakonen, M., Piitulainen, H., Visala, A., & Jämsä, T. (2015). A comparison of sEMG feature extraction methods for hand movement recognition in prosthetic control. Journal of Neuroengineering and Rehabilitation, 12(1), 24.

[19] Fougner, A., Scheme, E., Chan, A., Englehart, K., & Stavdahl, Ø. (2011). Resolving the limb position effect in myoelectric pattern recognition. IEEE Transactions on Neural Systems and Rehabilitation Engineering, 19(6), 644-651.

[20] Jiang, N., Dosen, S., Müller, K. R., & Farina, D. (2012). Myoelectric control of artificial limbs—is there a need to change focus? IEEE Signal Processing Magazine, 29(5), 150-152.

[21] Atzori, M., Gijsberts, A., & Caputo, B. (2012). The role of Electromyography in the design of human-robot interfaces for controlling a robotic hand. Proceedings of the IEEE, 100(8), 2406-2418..

[22] Arjunan, S. P., & Kumar, D. K. (2010). Comparison of ANN based classification techniques for sEMG signal identification. Journal of Medical Systems, 34(4), 647-656.

[23] Phinyomark, A., Nuidod, A., Phukpattaranont, P., & Limsakul, C. (2012). Application of wavelet analysis in EMG feature extraction for pattern classification. Measurement Science Review, 12(5), 247-253..

[24] Chowdhury, R. H., Reaz, M. B. I., Ali, M. A. M., Bakar, A. A. A., & Chellappan, K. (2013). Surface electromyography signal processing and classification techniques. Sensors, 13(9), 12431-12466.

[25] Radmand, A., Scheme, E., Englehart, K. B., & Hargrove, L. J. (2018). Effects of locomotion mode classification on the performance of simultaneous regression-based myoelectric control. IEEE Transactions on Neural Systems and Rehabilitation Engineering, 27(6), 1175-1186.
